# Identification of Aberrantly Methylated Differentially CpG Sites in Hepatocellular Carcinoma and Their Association With Patient Survival

**DOI:** 10.3389/fonc.2020.01031

**Published:** 2020-07-23

**Authors:** Renguo Guan, Weimin Guo, Weifeng Hong, Ye Lin, Xiongfeng Zou, Ning Shi, Dongyang Yang, Yu Zhou, Zhixiang Jian, Haosheng Jin, Weidong Lin, Min Yu

**Affiliations:** ^1^Department of General Surgery, Guangdong Provincial People's Hospital, Guangdong Academy of Medical Sciences, Guangzhou, China; ^2^Department of Pharmacy, Guangdong Provincial People's Hospital, Guangdong Academy of Medical Sciences, Guangzhou, China; ^3^Department of Medical Imaging, The First Affiliated Hospital of Guangdong Pharmaceutical University, Guangzhou, China; ^4^Department of Gastrointestinal Oncology, Cancer Center, Guangdong Provincial People's Hospital, Guangdong Academy of Medical Sciences, Guangzhou, China; ^5^Department of General Surgery, Affiliated Foshan Hospital of Southern Medical University, Foshan, China

**Keywords:** DNA methylation, hepatocellular carcinoma, survival, LBP, HBV

## Abstract

This study aimed to identify aberrantly methylated differentially methylated CpG sites (DMCs) and investigate their prognostic value in hepatocellular carcinoma (HCC). A total of 2,404 DMCs were selected from Gene Expression Omnibus (GEO) and validated by The Cancer Genome Atlas (TCGA). The TCGA cohort was divided into a training cohort and a validating cohort. First, the prognostic model based on six DMCs, including cg08351331, cg02910574, cg09947274, cg17589341, cg24652919, and cg26545968, was constructed based on the least absolute shrinkage and selection operator (LASSO) regression Cox analysis. The area under the curve (AUC) of the DMC-based model was 0.765 in the training cohort and 0.734 in the validating cohort. The accuracy of a model combining the DMC signature and American Joint Committee on Cancer (AJCC) stage, with an AUC of 0.795, was better than that of the DMCs or AJCC stage alone. Second, further analysis revealed that the methylation rate of cg08351331 was negatively associated with the expression of its relative gene, lipopolysaccharide-binding protein (LBP). Besides, the gene expression of LBP was significantly associated with poor overall survival in patients with hepatitis B virus (HBV) infection. Finally, these findings were confirmed by GSE57956 data and our own cohort. In conclusion, we established an accurate DMC-based prognostic model that could be combined with AJCC stage to improve the accuracy of prognostic prediction in HCC. Moreover, our preliminary data indicate that LBP may be a new key factor in HBV-induced HCC initiation through the regulation of its methylation.

## Introduction

Hepatocellular carcinoma (HCC), which accounts for ~85% of all primary liver cancers, is still one of the most fatal diseases worldwide (1). Viral infection, alcohol consumption, smoking, obesity, and gut-derived bacterial translocation have been proven to increase the risk of HCC. Patients with HCC are often confronted with a poor prognosis on account of a high rate of recurrence and metastasis, especially in patients who are diagnosed at a late stage (2). Although tremendous progress has been made in the development of cancer treatments, such as antiangiogenic targeted agents and checkpoint inhibitors, the treatment of HCC remains a challenge. Sadly, current evidence shows that these agents and inhibitors only subtly improve objective tumor response rates, with improvements ranging from 10 to 24%, which is not satisfactory (3, 4). Genetic and epigenetic alterations are common and involved in the pathogenesis of HCC. However, because the molecular mechanism of HCC varies, the prognosis of patients who are at the same stage may vary. Therefore, further elucidation of the molecular mechanism of HCC is still of great necessity for the development of novel therapeutic strategies.

Recently, with rapid development of biotechnology, multiple forms of “omics,” including genomics, epigenomics, transcriptomics, proteomics, and metabolomics, have facilitated the identification of molecular candidates with diagnostic value in HCC. DNA methylation, the addition of a group of three hydrogens and a carbon atom to CpG dinucleotides, is a process by which the expression of genes is repressed. Increasing evidence has indicated that it plays a pivotal role in several fundamental biological processes, such as organogenesis, X-chromosome inactivation, and genomic imprinting (5). Extensive evidence has demonstrated that altered DNA methylation in cancer cells can contribute to the repression of several genes involved in cellular functions, including cell cycle regulation (6). It has been shown that DNA methylation is considered one of the main mechanisms for the inactivation of tumor-associated suppressor genes that ultimately lead to carcinogenesis (7, 8). Recently, DNA methylation biomarkers have been used in clinical practice in many tumors, including colorectal cancer, renal cell carcinoma, and lung adenocarcinoma (9–12). As for HCC, it has been shown that the inactivation of tumor-associated suppressor genes followed by aberrant DNA methylation contributes to oncogenesis. Previous studies have confirmed that the inactivation of glutathione S-transferase pi 1 (GSTP1) gene expression caused by CpG island hypermethylation may be the potential mechanism underlying the pathogenesis of hepatitis B virus (HBV)–associated HCC (13, 14). Huang et al. (15) revealed that hypermethylation of ELF (embryonic liver fodrin), RASSF1A (Ras association domain family member 1), p16, and GSTP1 was associated with the progression of hepatocarcinogenesis. Other genes were found to be hypermethylated in HCC, serving as prognostic biomarkers, including VIM (vimentin) and FBLN1 (fibulin 1) (16). However, there are few studies investigating in depth the prognostic value of DNA methylation in HCC. Therefore, the present study aims to assess HCC-specific DNA methylation by analyzing the data downloaded from the Gene Expression Omnibus (GEO) database and The Cancer Genome Atlas (TCGA) and obtaining systematic information about their functions and their prognostic value in HCC.

## Materials and Methods

### DNA Methylation Data Selection From the GEO Database

The methylation dataset of eligible HCC datasets was searched in the GEO database. Datasets with more than 10 pairs of samples comparing the methylation profile between tumor and adjacent normal tissue were included in the present analysis. Only datasets with a definite diagnosis of HCC for all the included patients were considered. After carefully scrutinizing the GEO database, we found four datasets, including GSE54503 (17), GSE57956 (18), GSE37988 (19), and GSE73003 (20). GSE54503 was analyzed by using the HumanMethylation450 BeadChip, whereas the rest of them were analyzed by using the HumanMethylation27 BeadChip. GEO2R, which is based on the limma package (http://bioconductor.org/packages/limma/), was used to identify differentially methylated CpG sites (DMCs) between tumor samples and adjacent normal samples in these four datasets individually by analyzing the data, including the CpG site, associated genes, mean β value in tumors, mean β value in non-tumors, mean β value difference, and adjusted *P*-values. Values of *P* < 0.05 and log|FC| ≥ 0.1 were considered significant. Then, all of the DMCs from these four datasets were merged to identify the hypomethylated and hypermethylated CpG sites via the Venn tool. The annotation profile of DMCs was retrieved from the GPL8490 platform (Illumina HumanMethylation27 BeadChip). The distributions of hypermethylated and hypomethylated CpG sites relative to chromosomes, genes, and CpG islands were analyzed.

### Differentially Methylated CpG Sites Validated With TCGA Data

The raw data of these four datasets were downloaded from the GEO database and normalized by using the RMA algorithm from the limma package. The “SVA” package of R software was used to remove the batch effect and reduce the heterogeneity among these four datasets, and then each DMC obtained a unique fold change to validate the results from the GEO2R. Raw data including methylation (HM450) β values from the TCGA liver HCC (LIHC) cohort, which included 368 patients and 50 adjacent normal tissues, were also downloaded and processed using the Minfi package. Data filtering, correction, normalization, and quality control were implemented before analyzing the differentially methylated probes. Differentially methylated CpG sites of the LIHC cohort were also analyzed using the limma package to further validate the results from the GEO database. Volcano plots from the GEO and TCGA databases were conducted to identify the DMCs. Values of *P* < 0.05 and log|FC| ≥ 0.1 were considered significant.

### Functional and Pathway Enrichment Analysis

Functional annotation for the DMCs was performed by the bioinformatics tool “cluster Profiler” to comprehensively explore the functional relevance of these DMCs. Gene Ontology (GO) and Kyoto Encyclopedia of Genes and Genomes (KEGG) enrichment analysis were used to assess the functional categories related to the DMCs. Gene Ontology and KEGG terms with a *P*-value and *q* value of both <0.05 were considered significantly enriched categories. All statistical analyses were performed in R version 3.6.0 (the Statistics Department of the University of Auckland, New Zealand).

### Construction of the Prognosis Risk Model Based on DMCs

To explore the prognostic value of DMCs, the DMCs from the above analysis were used to build the prognostic risk model. To obtain an accurate prognostic model, we randomly divided the TCGA cohort into two cohorts of the equal size: the training cohort (*n* = 184) and the validating cohort (*n* = 184). For each DMC, we used the median value of the DMCs' level to divide the training cohort into two subgroups, and Cox univariate analysis were performed to identify survival-related DMCs (*P* < 0.05). Then, these survival-related DMCs were subjected to the least absolute shrinkage and selection operator (LASSO) regression Cox analysis (simulation times = 1,000) to identify a reliable DMC combination for HCC survival prediction. Next, we used the Glmnet package and survival package of R to build a risk score to calculate the coefficient and build a risk score model. The Glmnet package is built with the Cox regression algorithm, which is a semiparametric proportional risk model proposed by British biologist Dr. Cox (21). It is mainly used to analyze the influence of multiple factors on survival time at the same time. Its basic formula is as follows:

$$\begin{array}{l} h\left(t,X\right)/h_{0}\left(t\right)=\exp\left(\beta_{1}X_{1}+\beta_{2}X_{2}+\beta_{3}X_{3}+\ldots +\beta_{n}X_{n}\right) \end{array}$$

A prognosis risk prediction model based on β values and coefficients of six DMCs was established. Finally, the prognosis risk model was further validated in the validating cohort. To evaluate the prognostic value, we drew Kaplan–Meier (K-M) curves, and the cut-off value for significance was a *P* < 0.05. Receiver operating characteristic (ROC) curves were drawn, and the values of the AUC values were used to compare the predictive power of each model. All analyses were performed using R/Bioconductor (version 3.5.2).

### Association Between DMCs, Associated Gene Expression, and Methylation Status

The DMCs included in the prognostic risk model were further investigated to reveal the underlying molecular mechanisms. First, mRNA-seq data were downloaded from the LIHC TCGA dataset and submitted for analysis. All gene expression values were logarithmically transformed to approximate data to a normal distribution and then quantile normalized. The β value can be used to evaluate the methylation level: β value = methylated probe intensity/(methylated probe intensity + the unmethylated intensity +100) (22). Thus, we used the following cut-off values to determine the methylation level: 0.7–0.5 “hypermethylation” and 0.3–0.25 “hypomethylation” (23, 24). For genes with multiple probes, the mean β value of the probes of the same gene was denoted as the methylation status of genes for the LIHC cohort, which was different from the definition in the cBioPortal (http://www.cbioportal.org/). The associations between these variables were analyzed by Pearson correlation test, and *P* <0.05 was considered significant. The K-M estimator with a log-rank test was used to estimate the prognostic value of all these DMCs, associated gene expression and methylation status, and depicted in the forest plot.

### Association Between Lipopolysaccharide-Binding Protein and Its Methylation Sites

To reveal the underlying molecular mechanism of the prognostic risk model, we further explored the association between lipopolysaccharide-binding protein (LBP) and its multiple probes by MEXPRESS (https://mexpress.be/), which was developed to visualize DNA methylation and other profiles by utilizing TCGA data. We also analyzed associations between LBP expression and its multiple probes, as well as adjacent hepatic tissue inflammation, fibrosis score, histological type, sex, body mass index (BMI), and other factors.

The LIHC cohort was divided into two subgroups according to the median value of the LBP gene expression level, and functional and pathway enrichment analyses were performed between these two subgroups. Associations between LBP expression and pathological parameters were further analyzed based on the K-M plotter (https://kmplot.com/analysis/).

### Validation of LBP and cg08351331 in HCC Samples

To validate the association between LBP and cg08351331 and their prognostic role in HCC samples, we further analyzed 147 HCC samples from the General Surgery Department of Guangdong Provincial People's Hospital. All patients who donated their tissue samples for biomedical research, which were approved by the ethics committee of Guangdong Provincial People's Hospital, have signed informed consent forms. To detect the mRNA expression of LBP, quantitative reverse transcriptase–polymerase chain reaction (PCR) was performed as described previously (25). To achieve robust single-base specificity, a novel PCR-based method, named quantitative analysis of single-base methylation (QASM), was used to detect cg08351331 status in HCC samples (26). Briefly, genomic DNA was extracted using the QIAamp DNA Mini Kit (Qiagen, 51306, Hilden, Germany), and bisulfite was modified by using the EZ DNA Methylation Kit (Zymo Research, D5002, Orange County, California, USA). Then, the sodium bisulfite conversion of genomic DNA was amplified using locus-specific PCR primers flanking a pair of oligonucleotide probes, including a 5-fluorescent reporter dye 6-carboxyfluorescein (6FAM) or 2-chloro-7-phenyl-1,4-dichloro-6-carboxy-fluorescein (VIC), and a 3-quencher dye minor groove binder–non-fluorescent quencher (MGBNFQ). The MGB groups have a high sensitivity and specificity to single-base mismatch. The 5′-to-3′ nuclease activity of Taq DNA polymerase cleaves the probe and releases the reporter, whose fluorescence can be detected by the Applied Biosystems QuantStudio 7 Flex Real-Time PCR System, Shanghai, China. The methylation of each sample was equal to methylation/(methylated + unmethylated)/100, and the formula (100/(1 + 1/2^−ΔCT^), ΔCT = CT_methylated_ – CT_unmethylated_) was used to calculate the methylation rate. Values of *P* < 0.05 were considered significant. Primers for the present study were as follows: LBP forward: GAAGTTATATTTGTTGGTTGGATTTGG; LBP reverse: CCCACTCCCTATCCCCACTA; M probe: 6FAM-CCAAAACAACCTAAAACG-MGBNFQ; U probe: VIC-CCAAAACAACCTAAAATG-MGBNFQ. Statistical analysis was performed using the IBM SPSS Statistics software program version 22.0 (IBM Corp., Armonk, NY, USA).

## Results

### Identification of Aberrantly Methylated CpG Sites in LIHC

**Figure 1:**
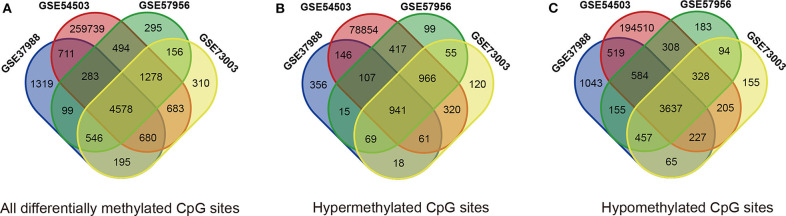
Identification of differentially methylated CpG sites (DMCs) in GEO datasets (GSE37988, GSE54503, GSE57956, and GSE73003). **(A)** All differentially methylated CpG sites; **(B)** hypermethylated methylated CpG sites; **(C)** hypomethylated methylated CpG sites.

**Figure 2:**
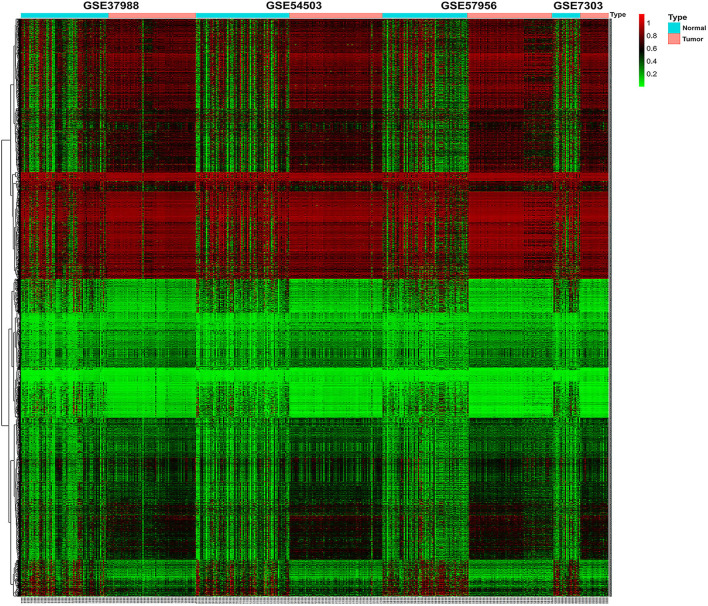
Heatmap of β values of the top 1,000 differentially methylated CpG sites in HCC patients and their corresponding adjacent normal samples in four GEO datasets. Rows represent differentially methylated CpG sites, and columns represent samples; light blue and pink represent normal and tumor tissues, respectively; green and red represent the low and high β values, respectively.

The methylation data from four datasets, including GSE54503, GSE57956, GSE37988, and GSE73003, were retrieved from the GEO database. There were 62 tumor samples and 62 paratumor samples in GSE37988; 66 tumor samples and 66 paratumor samples in GSE54503; 59 tumor samples and 59 paratumor samples in GSE57956; and 20 tumor samples and 20 paratumor samples in GSE73003. Detailed information about these four datasets is listed in Supplemental Tables 1, 2. In summary, a total of 207 tumor samples and 207 paratumor samples were included in this analysis. First, the *t*-test was performed to identify DMCs between tumor samples and adjacent normal samples in these four datasets individually. As depicted in Figure 1, numerous DMCs were found in these four datasets. Subsequently, these DMCs were merged to reveal a total of 4,578 overlapping DMCs, among which there were 3,637 hypomethylated DMCs and 941 hypermethylated DMCs. A heatmap of these DMCs is shown in Figure 2, which suggests that these four datasets differed slightly and may have a good consistency.

**Figure 3:**
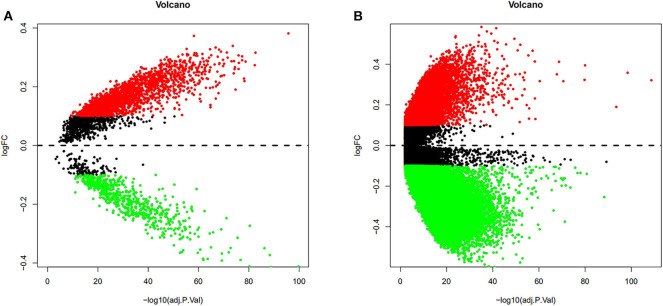
Volcano plots from the GEO and TCGA databases were conducted to depict the DMCs. Differentially methylated CpG sites from four GEO datasets were normalized, and the batch effects were removed. Among these 4,578 DMCs, only 3,737 DMCs were identified to significantly differ between tumor samples and adjacent normal tissues **(A)**. After analysis of the TCGA LIHC cohort between tumor samples and adjacent normal tissues, a total of 200,000 DMCs were found **(B)**. Values of *P* < 0.05 and log|FC| ≥ 0.1 were considered significant.

**Figure 4:**
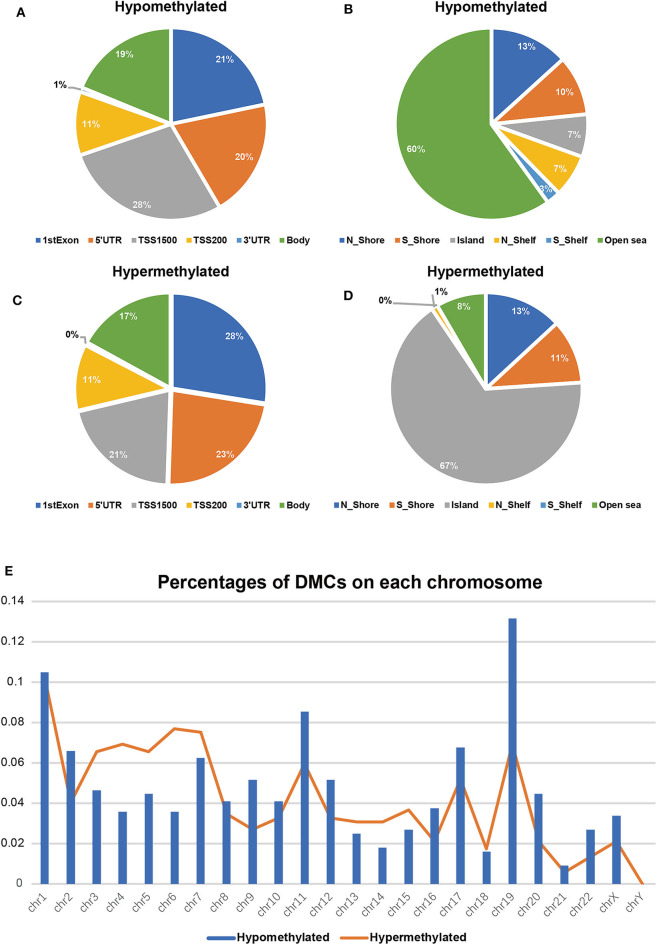
Differentially methylated CpG sites (DMCs) between HCC and adjacent normal tissues after overlapping with TCGA (*n* = 2,404). Hypomethylated CpG sites (*n* = 1832) relative to functional genomic distribution **(A)** and the CpG island along with its surrounding sequences **(B)**; hypermethylated CpG sites (*n* = 572) relative to functional genomic distribution **(C)** and the CpG island along with its surrounding sequences **(D)**. Location distribution in chromosomes between hypomethylated CpG sites (blue columns) and hypermethylated CpG sites (orange line) **(E)**.

To validate and analyze the DMCs between tumors and adjacent normal tissues, we first normalized the DMCs and removed the batch effects among the four datasets by using the SVA package. Among these 4,578 DMCs, only 3,737 DMCs were identified to significantly differ between tumor samples and adjacent normal tissues (Figure 3A). Further, the methylation data from the TCGA LIHC cohort were downloaded to confirm the present findings. After analysis of the TCGA LIHC cohort, a total of 200,000 DMCs were found (Figure 3B). Differentially methylated CpG sites from these four datasets were merged with 200,000 DMCs found in TCGA LIHC cohorts. The results showed that a total of 2,404 DMCs identified from these four datasets overlapped with the DMCs found in TCGA LIHC cohorts. We then investigated the location distribution of this 2,404 overlapping DMCs on CpG islands along with their surrounding sequences and functional genomic distribution (Figure 4). The results showed that ~23.8% (572 of 2,404) of overlapping DMCs were hypermethylated, whereas the rest of them were hypomethylated. Regarding the function of the genome, the distribution patterns of hypermethylated CpG sites were similar to those of hypomethylated CpG sites (*P* > 0.05). Similar to previous studies, the CpG islands can be divided into four subgroups according to their surrounding sequences: CpG island, shore, shelf, and open sea (27, 28). The results revealed that most hypomethylated DMCs were located in open sea regions (60% hypomethylated DMCs), whereas most hypermethylated DMCs were located in CpG islands (67% hypermethylated DMCs) (*P* < 0.0001). Of note, more hypermethylated CpG sites than hypomethylated CpG sites tend to be located at 1st exon (28% in hypermethylated CpG sites vs. 21% in hypomethylated CpG sites, *P* < 0.0001). Most of the hypomethylated and hypermethylated CpG sites were located at promoter regions, such as 1stExon, TSS1500, and others. Besides, we also explored the chromosome distribution of these 2,439 overlapping DMCs. As depicted in Figure 4, both hypomethylated and hypermethylated CpG sites occurred preferentially on chromosome 1 and chromosome 19.

### The Functional Annotation of DMCs

**Figure 5:**
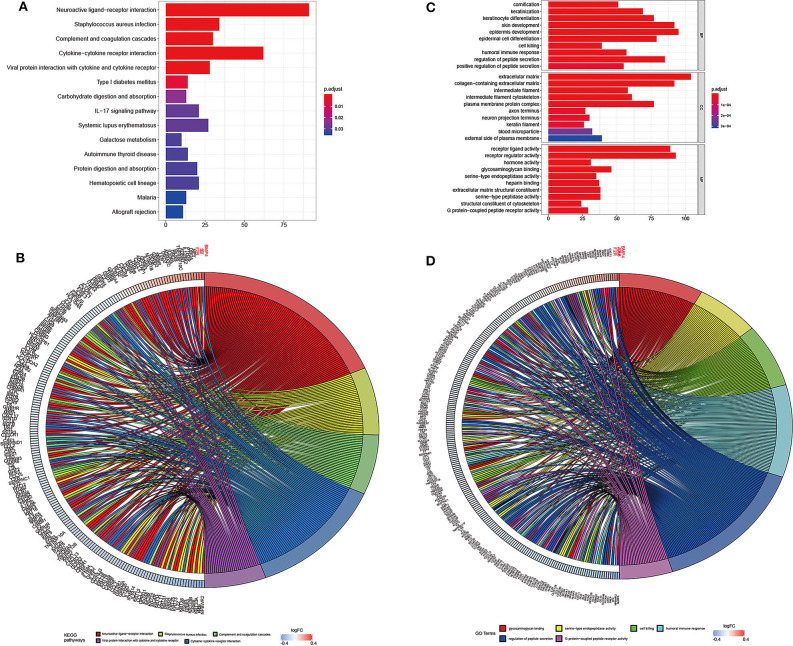
The top significantly enriched KEGG pathways and GO terms of associated genes for all the DMCs (*n* = 2,404) in HCC. The *x* axis shows the number of associated genes, and the *y* axis shows KEGG pathways **(A)** and GO terms **(B)**. Color changes as the adjusted *p* value changes. Chord plot depicting the associated genes for all the DMGs (*n* = 2,404) with ribbons to their assigned KEGG pathways **(C)** and GO terms **(D)**. Colored rectangles represent the log|FC| of genes.

The genes associated with DMCs were further analyzed by GO and KEGG pathway enrichment analysis. As depicted in Figure 5, glycosaminoglycan binding (*P* = 2.23E-08), serine-type endopeptidase activity (*P* = 3.48E-08), cell killing (*P* = 1.02E-08), humoral immune response (*P* = 1.05E-08), and regulation of peptide secretion (*P* = 1.08E-08) were the most significantly enriched GO terms. Of note, NLR family pyrin domain containing 3 (NLRP3) involved in the pathway of regulation of peptide secretion and glycosaminoglycan binding were significantly downregulated among genes with DMCs (*P* < 0.05). Bone morphogenetic protein 4 (BMP4), involved in the pathway of glycosaminoglycan binding, was the most upregulated gene (*P* < 0.05) in the GO enrichment analysis. KEGG pathway enrichment analysis revealed that neuroactive ligand–receptor interaction (*P* = 1.78E-17), *Staphylococcus aureus* infection (*P* = 1.08E-10), complement and coagulation cascades (*P* = 1.89E-10), and cytokine–cytokine receptor interaction (*P* = 1.16E-07) were significantly enriched pathways in HCC. Among DMCs associated with genes shown in the circle plot, NPBWR2 (neuropeptides B and W receptor 2), KRT36 (keratin 36), and SST (somatostatin) were the most significant genes in the pathway of neuroactive ligand–receptor interaction, whereas BMP4 and interleukin 22 (IL22) were the most significant genes in the pathway of cytokine–cytokine receptor interaction (*P* < 0.05).

### Predictive and Prognostic Value of the DMC-Based Signature

**Figure 6:**
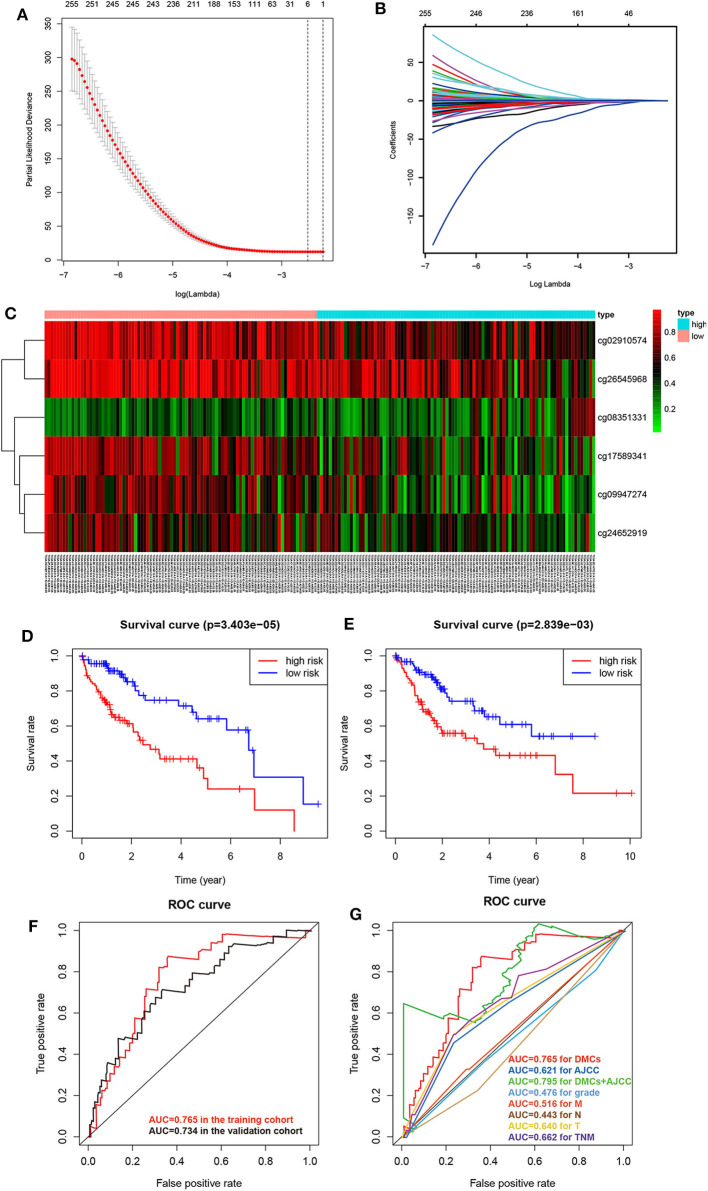
Establishment of the prognostic model of six DMCs in the training cohort (*n* = 183) from the LIHC cohort of TCGA. The cross-validation error curve shows the regularization path of the least absolute shrinkage and selection operator (LASSO) algorithm. The elimination of non-informative genes was visualized by decreasing prediction error to a minimum (black dotted line) with only the six most important DMCs left in the Cox proportional hazards model (gray shaded area = 5–95% confidence interval) **(A)**. LASSO regression analysis was performed to select radiomic features for prognostic model building for HCC patients. Feature coefficients were plotted against the shrinkage parameter (lambda) **(B)**. Heatmap of the six DMCs in the training cohort. Rows represent differentially methylated CpG sites, and columns represent samples; light blue and pink represent high and low β values of CpG sites, respectively **(C)**. To assess the prognostic value of the six-DMC model, overall survival was analyzed in the training cohort **(D)** and the validating cohort **(E)**. The red line and the blue line represent the patient groups with high- and low-risk scores, respectively. Receiver operating characteristic curve analysis was performed to assess the prognostic accuracy of the six-DMC model in the training cohort and the validating cohort **(F)**. The prognostic accuracy of the DMC model was further assessed by comparing it to other prognostic clinicopathological parameters, and the value of the AUC for each model is shown. Different colors represent different parameters and models, and the AUC values are depicted with the same color as the models **(G)**.

To obtain an accurate prognostic model, we randomly divided the TCGA cohort into two cohorts of the equal size, including the training cohort (*n* = 184) and the validating cohort (*n* = 184). Then, we used LASSO Cox regression to build a prognostic model, which identified six prognostic DMCs from the training cohort. The prognostic risk score was derived by a combination of β values and coefficients of all six DMCs (risk score = cg08351331 ^*^ 2.719 – cg02910574 ^*^ 1.129 – cg09947274 ^*^ 0.687 – cg17589341 ^*^ 1.043 – cg24652919 ^*^ 0.899 – cg26545968 ^*^ 0.752). We divided the training cohort into a high-risk subgroup and a low-risk subgroup according to the median value of prognostic risk scores (Figures 6A–C). Kaplan–Meier survival analysis suggested that the overall survival of patients in the low-risk subgroup was significantly better than that of patients in the high-risk subgroup in the training cohort (*P* = 3.403E-05). Following the above guideline, the validating cohort was divided into a high-risk subgroup and a low-risk subgroup as well, which revealed a significantly different overall survival of the two groups (*P* = 2.839E-03; Figures 6D,E). Receiver operating characteristic curves of the six DMCs were used to demonstrate the sensitivity and specificity in predicting the overall survival of patients. The results showed that the AUC of the six-DMC signature was 0.765 in the training cohort and 0.734 in the validating cohort, indicating that the prognostic model based on six DMCs had high sensitivity and specificity. We further compared this prognostic model with American Joint Committee on Cancer (AJCC), tumor node metastasis (TNM), and histological grade and found that the accuracy of the prognostic model based on six DMCs surpassed that of other clinicopathological parameters. Of note, the accuracy of a combined model based on DMCs and AJCC stage, with an AUC of 0.795, was better than that of DMCs or AJCC stage alone (Figures 6F,G). Therefore, it could be used to predict the prognosis of patients with LIHC with high accuracy. Moreover, the six-DMC signature could be combined with AJCC stage to improve the accuracy of prognostic prediction for HCC in the clinic.

### Relationship of Methylation, Gene Expression, and Prognosis

**Figure 7:**
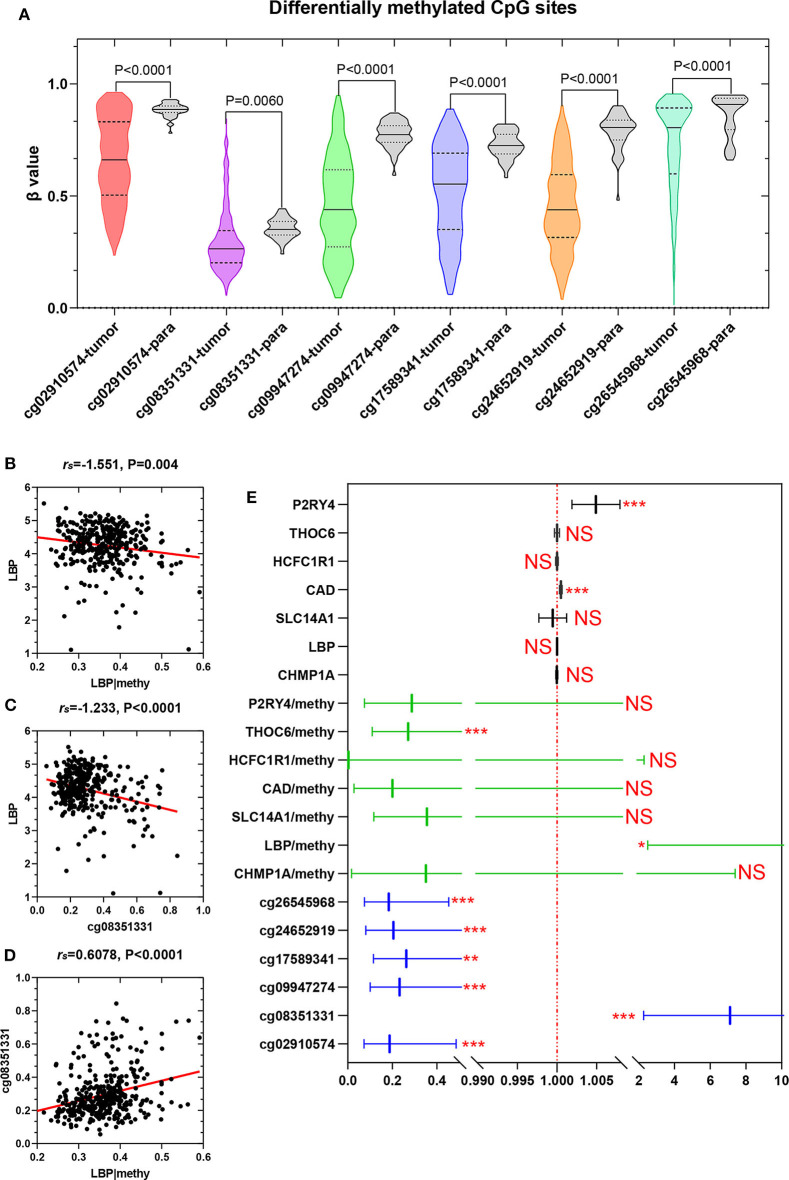
Expression of six DMCs between tumor tissues and adjacent normal tissues. The *x* axis shows each probe in the tumor (*n* = 368) and adjacent normal tissues (*n* = 50), and the *y* axis shows the β value of each probe in each sample. The detailed information of each probe of LBP is shown in the table. The Mann–Whitney *U*-test was used to determine the differences between the two groups, and *P*-values are shown above the violin plot **(A)**. The associations between LBP and its methylation status **(B)**, LBP and its probe cg08351331 **(C)**, and cg08351331 and LBP methylation status **(D)** are shown. The LBP methylation status was calculated as the mean methylation β value of all probes in the LIHC cohort (*n* = 368) from TCGA. A forest plot was used to depict the prognostic value for six DMCs, and the associated gene and methylation status were analyzed by univariate analysis. The black lines, green lines, and blue lines show the associated genes, methylation status, and six DMCs, respectively. ^*^P < 0.05, ^**^P < 0.01, and ^***^P < 0.001 **(E)**.

To elucidate the underlying relationship between the six DMCs and the survival of LIHC patients, we further individually investigated the prognostic value of these six DMCs in the whole cohort. Table 1 listed the six DMCs and their associated genes. We found that the levels of all six DMCs were significantly downregulated in LIHC tissues compared with paratumor tissues (all *P* < 0.05; Figure 7A). Then, the methylation values of each associated gene of these six DMCs were calculated to assess the whole methylation status of these genes. Correlation analyses were performed to assess the relationship between methylation and gene expression, and the prognostic value of these factors was also investigated (Supplemental Figure 1). The results showed that gene expression was closely correlated with gene methylation status in carbamoyl-phosphate synthetase 2, aspartate transcarbamylase, and dihydroorotase (CAD) (*r* = −0.598, *P* < 0.0001) and LBP (*r* = −1.551, *P* = 0.004; Figure 7B). The gene expression of LBP was significantly related to its corresponding cg08351331 β value (*r* = −1.233, *P* < 0.0001; Figure 7C). Similar results were also found for host cell factor C1 regulator 1 (HCFC1R1) (*r* = −0.2414, *P* = 0.0002) and THO complex 6 (THOC6) (*r* = −0.211, *P* < 0.0001). In addition, the β values of cg08351331 (*r* = 0.6078, *P* < 0.0001), cg24652919 (*r* = 1.365, *P* < 0.0001 for HCFC1R1; *r* = 0.252, *P* < 0.0001), and cg26545968 (*r* = 0.659, *P* < 0.0001) were significantly associated with increased methylation of associated genes (Figure 7D, Supplemental Figure 2). Therefore, the expression levels of associated genes, especially in LBP, HCFC1R1, and THOC6, were regulated at least partly by methylation. The above findings indicated that LBP expression was regulated by the total methylation status of LBP and even mainly controlled by cg08351331. Survival analysis revealed that all six DMCs were significantly associated with overall survival (all *P* < 0.001; Figure 7E). Of note, only the β value of cg08351331 had a negative association with overall survival, whereas other DMCs were closely associated with a better prognosis. The results suggested that the methylation status of THOC6 and LBP was closely correlated with overall survival (both *P* < 0.05). Moreover, only the gene expression of pyrimidineregic receptor P2Y4 (P2RY4) and CAD was significantly associated with prognosis (all *P* < 0.001). In summary, as shown in Figure 7, Supplemental Figures 1, 2, the results revealed that LBP expression and methylation were the most significantly changed between tumor and normal tissues. Surprisingly, the gene expression of LBP was not significantly associated with overall survival (*P* > 0.05), although its methylation status and promoter probe cg08351331 β value predicted poor prognosis in LIHC.

**Table 1:** The detailed information of the six DMCs according to HumanMethylation450.

**Name**	**Forward_sequence**	**Chromosome location**	Strand^*^	**Reference Gene**	**Relation to gene content**	**Relation to CpG island**
cg02910574	AAAGTAGGAAGAAGCTAGAAT ACAAAGTTTTCCATCAGTTTT ACCACGTGGAGCTGGAAG [CG]TTAAATATTTTAAACAACG GAGGTGCTGCCTGGGGCG GTAGTTCACACCTGTCCTCCC AG	16	F	CHMP1A	TSS1500	S_Shore
cg08351331	CCGACAAGGGACTGCAGTATG GTAAGAAGCCACATCTGCTGG CTGGACTTGGCAAACCCA[CG] CTCCAGGCTGCTCTGGGTA CAGTGGGGACAGGGAGTG GGGACACAGACCGGACCC TCTCC	20	R	LBP	Body	_
cg09947274	ACTATCTTAGCCCAGTCAG GGAGCTCTGCTTCCTAGAAAG GCATCTTTCGCCAGTGGA TT[CG]CCTCAAGGTTGAGGC CGCCATTGGAAGATGAAAAAT TGCACTCCCTTGGTGTAGACA AAT	2	R	CAD	3′UTR	N_Shore
cg17589341	CCAGGGGACCAGTTCCTTGGT GTTGCTTTGGCATTGATGCCT GAAGTGGGAGGAGAAAGC [CG]AGCCCACAAACACACAG AGCAGAGTGGGGCTCTGAGTA TATAACTGTTAGGTGCCTCCCT	18	F	SLC14A1	TSS200;TSS200;TSS200	_
cg24652919	GAGGTCAGCTAGAGCTAAAAA TGGCACGATGAGGCAGGG CCTGAGGTCAGCTGTGAGGAC [CG]CCTGTGATTCTGCAGAA GGCCTGGCCAGTGGAGGAA CCTACCTGAGTGGGGGCA GGGCTG	16	R	HCFC1R1;THOC6	Body;Body;Body;TSS1500; TSS1500	N_Shore
cg26545968	GCCCTTTACGATGACCTAGTC CAACCCACTCATTGTACAGAT TGGGAAACTGGGGCCCAG [CG]AGATTACTATTTAACAGCTT TGAAATGACTGCAGGAGGGA GAACACGAGGAAACATCCAA	X	F	P2RY4	TSS1500	_

* *F, forward stand; R, reverse stand*.

### The Prognostic Significance of LBP Is Dependent on the Hepatitis Virus Infection

**Figure 8:**
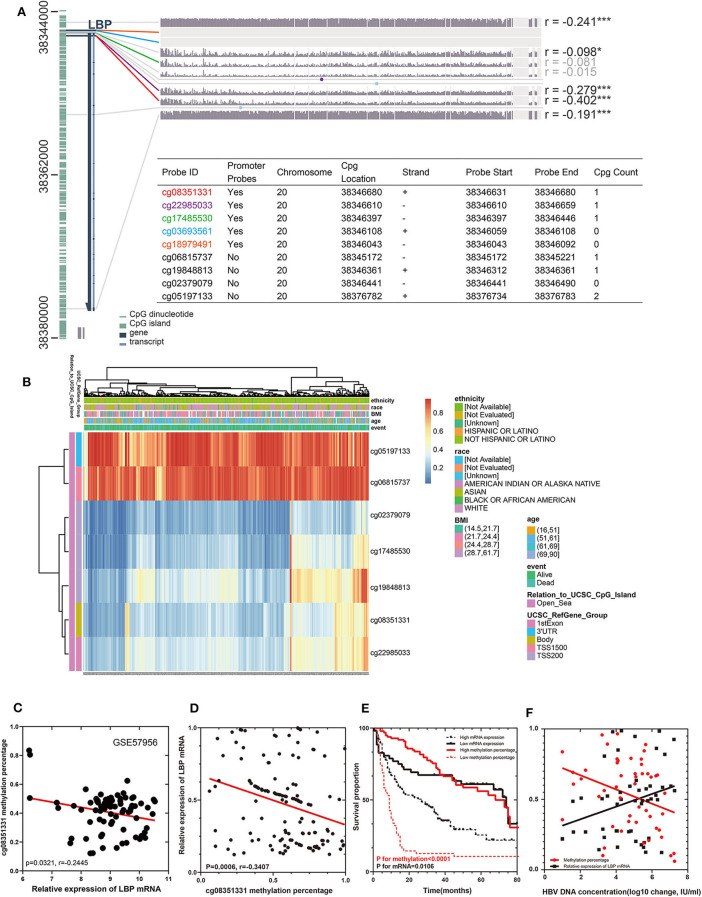
Visualization of TCGA data (*n* = 368) and GSE57956 data for LBP in HCC. The left aquamarine column represents the sequence of LBP, and each probe is marked in the sequence. The associations between the methylation β value of each probe and LBP gene expression are shown. The numbers on the far right indicate the significance of the correlation (Pearson correlation coefficient) ^*^P < 0.05, ^**^P < 0.01, and ^***^P < 0.001. **(A)**. The expression of the seven probes of LBP with different clinicopathological features is depicted **(B)**. The relationship between the methylation rate of cg08351331 and the relative expression of LBP mRNA in GSE57956 **(C)**. A cohort of 147 HCC samples and 10 normal liver samples was included to validate the findings from GEO datasets and TCGA cohorts. A novel technique (QASM) was used to assess the methylation rate of cg08351331, and the mean methylation value of 10 normal samples was used as a control. Reverse transcriptase–PCR was used to examine the expression of LBP mRNA, whereas the concentration of HBV DNA was assessed by fluorescence quantitative PCR. The correlations among cg08351331, LBP mRNA, and HBV DNA concentration were analyzed by Pearson correlation test **(D,F)**. Survival analysis was performed to compare patients with high LBP mRNA and low LBP mRNA levels and those with high cg08351331 methylation rate and low cg08351331 methylation rate **(E)**.

To elucidate the discrepancy in the prognostic roles of LBP and its probes in LIHC, we first investigated the relationship between LBP and its probes. Given the close relationship between cg08351331 and LBP, we suspected that cg08351331 may regulate LBP gene expression. We explored the potential methylation probes on both forward and reverse strands on the LBP sequence using the MEXPRESS tool (https://mexpress.be/) by analyzing the LIHC cohort from TCGA. As indicated in Figure 8A, there were nine methylation probes on both strands, five of which were promoter probes, including cg08351331, cg22985033, cg17485530, cg03693561, and cg18979491. However, two probes, cg03693561 and cg18979491, were not detected in the HM450 array. Notably, the β values of the promoter probes cg08351331 (*r* = −0.402, *P* < 0.001) and cg22985033 (*r* = −0.279, *P* < 0.001) were significantly associated with LBP gene expression (https://biit.cs.ut.ee/methsurv/) (Figure 8B). Considering that promoter-associated hypermethylation can trigger transcriptional silencing of the target gene, decreased methylation of cg08351331 in LIHC may reduce methylation of the LBP gene promoter, thus increasing LBP gene expression.

We further divided the LIHC cohort into high expression and low expression groups according to the median value of the LBP gene expression level and analyzed the differentially expressed genes between these two groups. Gene Ontology and KEGG analysis suggested that these genes clustered in acute-phase response and inflammatory response (*P* < 0.01), which implied that LBP in live cancer cells may engage in the local immune response to gut-derived bacterial translocation, which was an etiological factor of HCC according to current reports (29, 30). The association of LBP and clinicopathological parameters was further investigated by analyzing the LIHC cohort from TCGA. As indicated in Supplemental Figure 3, LBP gene expression was closely associated with patient age at the initial pathologic diagnosis (*r* = 0.107, *P* < 0.05), which was consistent with the previous finding (31). Interestingly, the results suggested that LBP gene expression was significantly associated with the BMI status of patients (*r* = 0.178, *P* < 0.001), which was found to be significantly associated with cancer prognosis (32–34). In summary, LBP may engage in the development and progression of HCC, and the prognostic value of LBP needs further elucidation. Then, we divided patients from the LIHC cohort into several subgroups to identify the reasons for prognostic discrepancy according to the clinicopathological parameters. The results in Table 2 indicated that the gene expression of LBP predicted poorer overall survival in the early stage (stage 1 + 2) than in the late stage (stage 3 + 4) (*P* = 0.0062). Gene expression of LBP was significantly associated with poorer overall survival in AJCC T1 (*P* = 0.0024) but better overall survival in AJCC T2 (*P* = 0.0288). Interestingly, the gene expression of LBP was significantly associated with poorer overall survival in patients with hepatitis virus infection (*P* = 0.005) but better overall survival in patients without hepatitis virus infection (*P* = 0.0066). Thus, it seems that the function and prognostic value of LBP in HCC are related to the status of hepatitis virus infection.

**Table 2:** Prognostic value of LBP with different clinicopathological factors in the LIHC cohort of TCGA from Kaplan-Meier plotter database.

		**OS (*****n*** **=** **364)**			**RFS (*****n*** **=** **316)**		
		**Patient number**	***P*-value**	**HR [95% CI]**	**Patient number**	***P*-value**	**HR [95% CI]**
**Pathology**							
**Stage**							
	1 + 2	253	0.0062	3.2 (1.33–7.69)	227	0.4262	1.2 (0.77–1.88)
	3 + 4	87	0.0573	0.56 (0.31–1.03)	68		
**Grade**							
	1	55			45		
	2	174	0.0981	1.57 (0.92–2.68)	147	0.3715	1.25 (0.77–2.03)
	3	118	0.104	1.65 (0.9–3.02)	106	0.1012	0.63 (0.37–1.1)
	4	12					
**AJCC_T**							
	1	180	0.024	1.97 (1.08–3.58)	160	0.3479	1.31 (0.75–2.29)
	2	90	0.0288	0.44 (0.21–0.94)	79		
	3	78			65		
	4	13			6		
**Vascular invasion**							
	None	203	0.1673	1.43 (0.86–2.4)	175	0.1106	1.49 (0.91–2.45)
	Micro	90	0.0451	2.37 (0.99–5.64)	81	0.3381	0.72 (0.36–1.42)
	Macro	16			14		
**Patient**							
**Gender**							
	Male	246	0.124	1.42 (0.91–2.21)	208	0.044	0.64 (0.41–0.99)
	Female	118	0.0925	0.6 (0.33–1.09)	105	0.2189	1.47 (0.79–2.75)
**Race**							
	White	181	0.216	0.72 (0.43–1.21)	147	0.1142	0.67 (0.41–1.1)
	Black or African American	17			13		
	Asian	155	0.0508	1.83 (0.99–3.38)	143	0.1677	0.69 (0.4–1.17)
**Sorafenib treatment**							
	Treated	30			22		
**Risk factors**							
**Alcohol consumption**							
	Yes	115	0.1001	0.57 (0.29–1.12)	98	0.1184	0.61 (0.32–1.14)
	No	202	0.0332	1.65 (1.04–2.62)	183	0.2877	0.77 (0.47–1.25)
**Hepatitis virus**							
	Yes	150	0.005	2.45 (1.28–4.67)	138	0.2678	0.74 (0.44–1.26)
	No	167	0.0066	0.52 (0.32–0.84)	143	0.1187	0.66 (0.39–1.12)

*Cohorts patients only more than 80 were analyzed further*.

We utilized GSE57956 data and our cohort containing 147 HCC samples from Guangdong Provincial People's Hospital to validate these findings from the LIHC cohort. Notably, as shown in Figure 8C, the methylation rate of cg08351331 was negatively significantly associated with the relative expression of LBP mRNA (*P* = 0.0321, *r* = −0.2445). The clinical and pathological characteristics of these patients are shown in Supplemental Table 3. As shown in Supplemental Table 4, univariate Cox analysis and multivariate Cox hazards analysis revealed that the expression of cg08351331, performance status, the level of albumin, and the existence of vascular cancer embolus were independent risk factors in our cohort (all *P* < 0.05). The results of QASM indicated that the methylation rate of cg08351331 was negatively and significantly associated with the relative expression of LBP mRNA (*P* = 0.006, *r* = −0.3407; Figure 8D). Moreover, a low methylation rate of cg08351331 and high expression levels of mRNA were significantly associated with poorer overall survival (*P* for methylation <0.0001, *P* for mRNA = 0.0106; Figure 8E). As almost all of the patients included in the present study were infected with HBV, we further investigated the role of HBV infection in LBP methylation and expression. The results suggested that the methylation rate of cg08351331 was negatively associated with HBV DNA concentration (*P* = 0.0219, *r* = −0.1047), whereas the relative expression of LBP mRNA was positively associated with HBV DNA concentration (*P* = 0.0464, *r* = 0.08011; Figure 8F). Our results further confirmed the relationship between virus infection and LBP expression found in TCGA and GEO datasets. Therefore, our preliminary data indicate that LBP may be another key element by which virus infection leads to the development and progression of HBV-related HCC.

## Discussion

Hepatocellular carcinoma, accounting for 85% of primary liver cancers, is still one of the most common malignant cancers in the world (35). According to the report of CONCORD-3, which includes data from 322 population-based cancer registries of 71 countries and regions, the survival rate of liver cancer in a few countries has increased by 5% (36), highlighting the need to develop novel therapeutic approaches. Fortunately, with the help of microarray and high-throughput sequencing technologies, it is possible to investigate epigenetic alterations that affect tumorigenesis and to identify potential biomarkers that offer strategies for liver cancer management. DNA methylation is one of the main reasons for the silencing of tumor-associated suppressor genes that ultimately contributes to carcinogenesis. Thus, DNA methylation provides us with a novel direction for cancer treatment. In this study, we performed a genome-wide DNA methylation analysis to identify ideal prognostic biomarkers and search for a novel target for cancer therapy. A total of 207 tumor samples and 207 paratumor samples from GSE54503, GSE57956, GSE37988, and GSE73003 were used to identify DMCs in LIHC. There were 4,589 overlapping DMCs extracted, among which 2,404 DMCs were further validated with the TCGA LIHC cohorts. According to the CpG island and its surrounding sequences, we found that the distribution pattern of hypermethylated CpG sites in relation to CpG islands was similar to that of hypomethylated CpG sites. The results indicated that most of the DMCs were hypomethylated, which was consistent with a previous report (37).

A previous report indicated that hypermethylation of gene body CGIs was associated with gene upregulation in 56% of HCC patients, who belong to the “HCC proliferative-progenitor” subclass, indicating elevated oncogene levels in HCC (38). Moreover, we found that hypomethylated CpG sites and hypermethylated CpG sites were located on the gene body at similar frequencies in the present study, indicating that not only hypermethylated but also hypomethylated CpG sites may also be involved in the oncogenesis of HCC. Given that promoter-associated methylation can modulate the expression of genes (39), it was reasonable that most of the hypomethylated and hypermethylated CpG sites were located at promoter regions, such as 1st exon, TSS1500, and others. The GO and KEGG pathway enrichment analyses were conducted to expound the potential biological functions of DMCs in the development of HCC. Glycosaminoglycan binding, serine-type endopeptidase activity, cell killing, humoral immune response, and regulation of peptide secretion were the most significantly enriched GO terms, whereas ligand–receptor interaction, *S. aureus* infection, complement and coagulation cascades, and cytokine–cytokine receptor interaction were significantly enriched in the KEGG pathway analysis. Of note, NLRP3, which is involved in the pathway of regulation of peptide secretion and glycosaminoglycan binding, was the most significantly downregulated among genes with DMCs. A previous study showed that NLRP3 inflammasome activation was inhibited by HBV infection, and this phenomenon existed in patients with HBV-related HCC. Further study indicated that HBV infection can inhibit the activation of the NLRP3 inflammasome pathway, thus reducing the production of proinflammatory factors, which enable virus immune escape from the local immune response (40). It is worth noting that our results suggest that NLRP3-related cg21991396 was significantly downregulated in HCC, which was positively associated with the expression of NLRP3. Of note, cg21991396 is located in the gene body rather than the promoter region of NLRP3, which may facilitate increased NLRP3 expression. Taking all the results into account, we speculated that HBV infection may inhibit the activation of the NLRP3 inflammasome pathway through the regulation of NLRP3 methylation and thus be involved in the oncogenesis of HCC. BMP4, whose gene body CpG site cg14310034 was significantly upregulated and involved in the pathway of glycosaminoglycan binding, was the most upregulated gene in the GO enrichment analysis, indicating the oncogenic role of BMP4 in HCC. A previous report suggested that BMP4 can activate autophagy through c-Jun amino-terminal kinase 1–mediated Bcl-2 phosphorylation, thus promoting HCC proliferation, which was consistent with our result (41).

Additionally, a prognostic model with six DMCs, including cg08351331, cg02910574, cg09947274, cg17589341, cg24652919, and cg26545968, was constructed, and its prognostic risk score was constructed as well. Thus, patients could be divided into a high-risk subgroup and a low-risk subgroup based on the median of prognostic risk score as a threshold. In our study, K-M overall survival curve in the low-risk group and the high-risk group was significantly different. Our prognostic model showed high accuracy in predicting the prognosis of HCC. In clinical practice, we can use this prognostic model to identify patients with different risk scores, which will be treated with different strategies. For those patients with high-risk scores, we should strengthen post-operative monitoring and follow-up and take more active treatment strategies, such as targeted therapy and so on. For those patients with poor prognosis, we should avoid unnecessary surgical operation to relieve their pain, and at this time, our dominating goal is to improve the quality of life. Moreover, the models combining the AJCC stages and six DMCs had higher AUCs than those based on the AJCC stages alone or six DMCs alone. Thus, we suppose that, in clinical practice, the six-DMC signature could be combined with AJCC stage to improve the accuracy of prognostic prediction in HCC. Further, we investigated the six DMCs to provide valuable drug targets for HCC in the future. Our results suggested that there were significant negative associations between LBP gene expression and its promoter probe cg08351331, implying that promoter-associated hypermethylation can trigger transcriptional silencing of the target gene and *vice versa*. A recent study found that LBP, synthesized in the liver and secreted into circulation constitutively, is of great significance for endotoxin recognition, presentation, and subsequent cytokine induction in the acute-phase response (42). Further studies also revealed that LBP in exosomes was able to effectively distinguish between patients with metastatic and patients with non-metastatic non-small cell lung cancers, but the underlying mechanism is still unknown (43). Some studies found that LBP expression predicted a high risk of post-operative progression in conventional renal cell carcinoma (44). However, a large nested case-control study, including 1,638 participants (819 colorectal cancer cases and 819 well-matched controls), found that LBP (a marker of lipopolysaccharide exposure) was not significantly associated with the overall survival of colorectal cancer patients. A previous study suggested that LBP in HCC cells engaged in the release of inflammatory mediators, behaving like a type 1 acute phase protein (45). No studies concerning the prognostic value of LBP in HCC have been previously conducted. Therefore, we conducted the present study to investigate the prognostic value of LBP in HCC.

Further analysis revealed that the gene expression of LBP was not significantly associated with overall survival, whereas its methylation status and promoter probe cg08351331 β value predicted poor prognosis in LIHC. We further found that LBP may engage in the local immune response to gut-derived bacterial translocation, which is an etiological factor in HCC according to current reports (29, 30). The results also indicated that LBP gene expression was significantly associated with the BMI status of patients from the LIHC cohort, which was found to be significantly associated with cancer prognosis (32–34). Therefore, it is reasonable to expect the prognostic value of LBP. To elucidate the discrepancy, we split the LIHC cohort according to clinicopathological parameters. Surprisingly, the gene expression of LBP was significantly associated with poor overall survival in patients with hepatitis virus infection and good overall survival in patients without hepatitis virus infection. The results suggested that the function and prognostic value of LBP in HCC depended on the status of hepatitis virus infection. The dual roles of LBP also remind us that it is necessary to assess the function of genes under the same baseline conditions. To further confirm the above results, a cohort of samples from our department was enrolled. The results suggested that there was an inverse correlation between the methylation rate of cg08351331 and HBV DNA concentration, whereas the relative expression of LBP mRNA was positively associated with HBV DNA concentration. Moreover, a low methylation rate of cg08351331 and high expression levels of mRNA were significantly associated with poorer overall survival. Therefore, our results further confirmed the relationship between virus infection and LBP expression found in the TCGA and GEO datasets, which suggested that gene expression of LBP was significantly associated with poor overall survival in patients with hepatitis virus infection. LBP is the binding protein of lipopolysaccharide. Most studies concerned the acute-phase response of LBP to bacterial infection (46–48); few studies focused on the role of LBP in virus infection, and the underlying mechanisms of LBP remain unclear. Of note, a previous study showed that people infected with HIV had a significantly higher level of LBP than uninfected individuals (49). There was also a study indicating that patients with hepatitis C virus (HCV) infection had significantly elevated LBP compared to those without HCV infection (50). It was reported that, as the composition of outer cell wall of gram-negative bacteria, lipopolysaccharide could stimulate Kupffer cells and hepatic stellate cells in the liver, thereby promoting the progress of liver fibrosis (51). In our study, there was a positive correlation between LBP mRNA and HBV DNA concentration. Our preliminary data indicate that LBP may be another key element in the development and progression of HBV-related HCC through the regulation of LBP methylation. We suppose that the combination of anti-HBV drugs and drugs that target LBP or cg08351331 may improve the prognosis of HCC patients. However, the underlying mechanism by which viruses engage in the regulation of LBP methylation is still unknown and needs further exploration.

Our study has some limitations. First, the data for the four datasets and the TCGA cohort were not acquired using the same platforms, which indicates that combining all these data inevitably ignored some important information. Second, HCC patients without HBV infection were scarce in our department because of the prevalence of HBV infection, and we were unable to validate the favorable prognostic value of LBP in HCC patients without HBV infection by using our data. Third, further studies are needed to verify the biological function of the other five DMCs and their associated genes.

In summary, we established a prognosis risk model based on six DMCs that could be combined with the TNM stage to improve the accuracy of prognostic prediction in HCC. Further, our preliminary data indicate that LBP, one of the associated genes of the model, may be a new key factor that mediates HBV-induced HCC initiation through the regulation of LBP methylation. More clinical studies on the functional mechanism of the six-DMC signature should be examined to determine its role in carcinogenesis.

## Data Availability Statement

The datasets analyzed in this study can be found in the Cancer Genome Atlas (https://tcgadata.nci.nih.gov/tcga/); the NCBI Gene Expression Omnibus (GSE54503, GSE57956, GSE37988, and GSE73003).

## Author Contributions

RG, WG, and WH contributed equally to the development of methodology, acquisition of data analysis and interpretation of data, and writing, review, and revision of the manuscript. XZ and NS contributed to administrative, technical, or material support. DY contributed to study supervision. ZJ and YZ contributed to revision of the manuscript. MY, HJ, and WL contributed to conception and design. All authors participated in the discussion and editing of the manuscript.

## Conflict of Interest

The authors declare that the research was conducted in the absence of any commercial or financial relationships that could be construed as a potential conflict of interest.

## Supplementary Material

Supplemental Figure 1 The associations between the five DMCs, their associated genes and their methylation status, except LBP and its methylation site cg08351331. The methylation status of associated genes was calculated by using the mean methylation β value of all probes in the associated genes from the LIHC cohort (*n* = 368) of TCGA. The correlations among them were analyzed by the Pearson correlation test. **(A)** The associations between the CHMP1A and its methylation status. **(B)** The associations between the CHMP1A and cg02910574. **(C)** The associations between the methylation status of CHMP1A and cg02910574. **(D)** The associations between the SLC14A1 and its methylation status. **(E)** The associations between the SLC14A1 and cg17589341. **(F)** The associations between the methylation status of SLC14A1 and cg02910574. **(G)** The associations between the CAD and its methylation status. **(H)** The associations between the CAD and cg09947274. **(I)** The associations between the methylation status of CAD and cg09947274. **(J)** The associations between the HCFC1R1 and its methylation status. **(K)** The associations between the HCFC1R1 and cg24652919. **(L)** The associations between the methylation status of HCFC1R1 and cg24652919. **(M)** The associations between the THOC6 and its methylation status. **(N)** The associations between the THOC6 and cg24652919. **(O)** The associations between the methylation status of THOC6 and cg24652919. **(P)** The associations between the P2RY4 and its methylation status. **(Q)** The associations between the P2RY4 and cg26545968. **(R)** The associations between the methylation status of P2RY4 and cg26545968. ^*^*P* < 0.05, ^**^*P* < 0.01, and ^***^*P* < 0.001.

Supplemental Figure 2 Survival analysis was performed to explore the prognostic value of DMCs, their associated genes, and their methylation status; Kaplan–Meier survival curves for each analysis are shown (*n* = 368 from the LIHC cohort of TCGA).

Supplemental Figure 3 Patients from the TCGA cohort were divided into two groups according to the expression of LBP mRNA, and the genes that differed significantly between these two groups are depicted in the heatmap. Rows represent differentially expressed genes, and columns represent samples; green and red colors represent the low and high logFC of gene expression, respectively. Values of *P* < 0.05 were considered significant **(A)**. The top significantly enriched KEGG pathways and GO terms of differentially expressed genes in HCC. The inner circle represents the logFC of differentially expressed genes, and the red and blue colors represent the high and low logFC values for gene expression, respectively. The outer circle represents the GO terms **(B)** and KEGG pathways **(C)** associated with the differentially expressed genes. The expression of LBP mRNA with different clinicopathological features is depicted **(D)**. The numbers on the far right indicate the significance of the correlation (correlation coefficient or *P*-value, depending on the data types compared) between each row of data (clinical characteristics, expression). ^*^*P* < 0.05, ^**^*P* < 0.01, and ^***^*P* < 0.001.

Supplemental Table 1 GEO series used in our study.

Supplemental Table 2 Clinical and pathological characteristics of HCC patients in GSE54503, GSE57956, GSE37988, and GSE73003.

Supplemental Table 3 Clinical and pathological characteristics of 147 HCC patients.

Supplemental Table 4 Prognostic factors for overall survival (univariate and stepwise multivariate Cox hazard analysis).

## Abbreviations

HCC: hepatocellular carcinoma
TCGA: The Cancer Genome Atlas
DMCs: differentially methylated CpG sites
AJCC: American Joint Committee on Cancer
TNM: tumor node metastasis
GO: Gene Ontology
KEGG: the Kyoto Encyclopedia of Genes and Genomes
ROC: receiver-operator characteristic
AUC: area under the curves
LIHC: liver hepatocellular carcinoma
LBP: lipopolysaccharide binding protein
GSTP1: glutathione S-transferase pi 1
ELF: embryonic liver fodrin
RASSF1A: Ras association domain family member 1
VIM: vimentin
FBLN1: fibulin 1
GEO: Gene Expression Omnibus
NLRP3: NLR family, pyrin domain containing 3
BMP4: bone morphogenetic protein 4
NPBWR2: neuropeptides B and W receptor 2
KRT36: keratin 36
SST: somatostatin
CAD: carbamoyl-phosphate synthetase 2, aspartate transcarbamylase, and dihydroorotase
HCFC1R1: host cell factor C1 regulator 1
THOC6: THO complex 6
P2RY4: pyrimidineregic receptor P2Y4
QASM: quantitative analysis of single-base methylation
HBV: hepatitis B virus
HCV: hepatitis C virus
LASSO: least absolute shrinkage and selection operator
BMI: body mass index.
