# Supplementary figures and images for: Identification of Aberrantly Methylated Differentially CpG Sites in Hepatocellular Carcinoma and Their Association With Patient Survival
Source: Front Oncol. 2020 Jul 23;10:1031. doi: 10.3389/fonc.2020.01031 (PMC7390903; doi:10.3389/fonc.2020.01031)

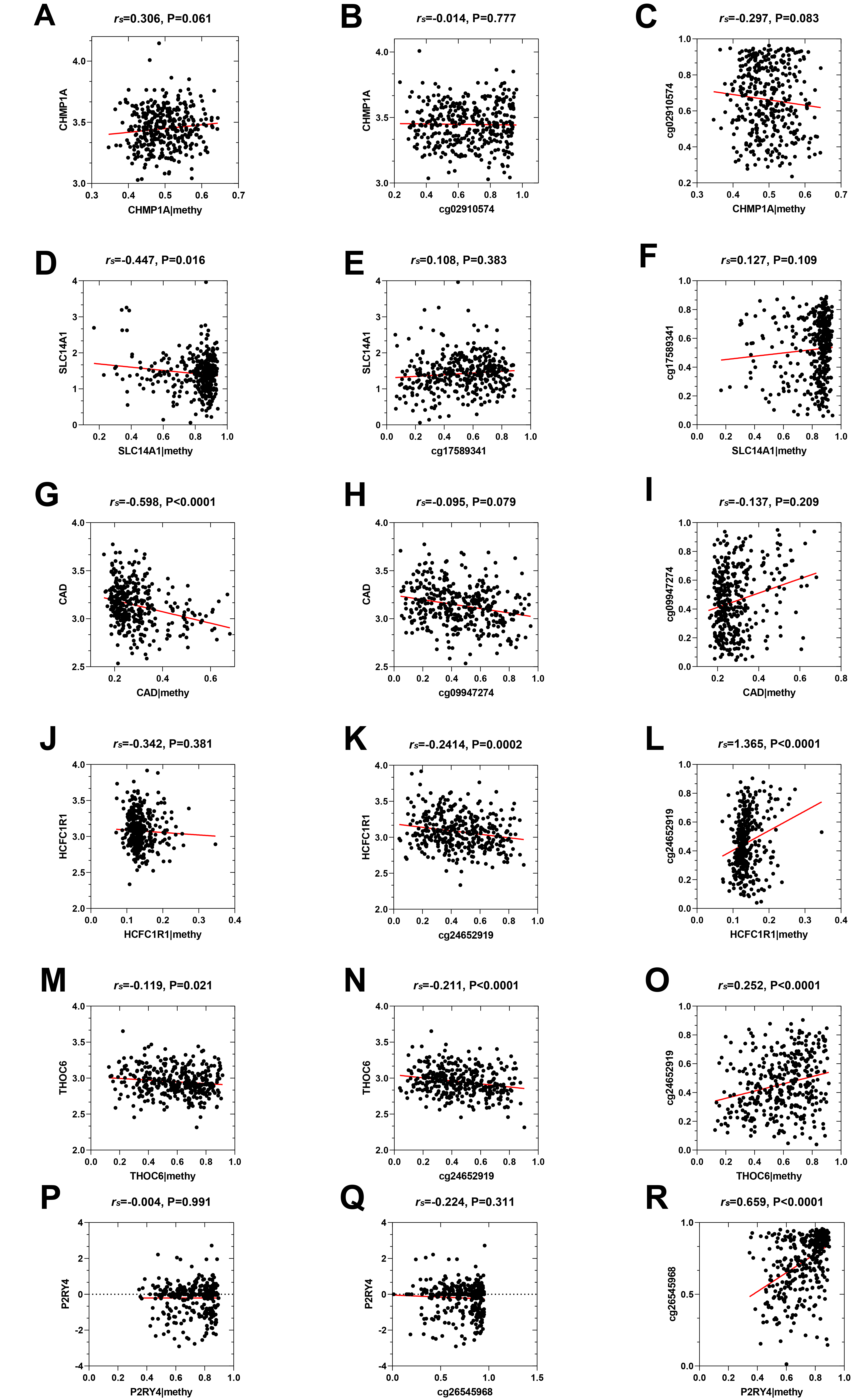

Supplement: Supplemental Figure 1 — The associations between the five DMCs, their associated genes and their methylation status, except LBP and its methylation site cg08351331. The methylation status of associated genes was calculated by using the mean methylation β value of all probes in the associated genes from the LIHC cohort (n = 368) of TCGA. The correlations among them were analyzed by the Pearson correlation test. (A) The associations between the CHMP1A and its methylation status. (B) The associations between the CHMP1A and cg02910574. (C) The associations between the methylation status of CHMP1A and cg02910574. (D) The associations between the SLC14A1 and its methylation status. (E) The associations between the SLC14A1 and cg17589341. (F) The associations between the methylation status of SLC14A1 and cg02910574. (G) The associations between the CAD and its methylation status. (H) The associations between the CAD and cg09947274. (I) The associations between the methylation status of CAD and cg09947274. (J) The associations between the HCFC1R1 and its methylation status. (K) The associations between the HCFC1R1 and cg24652919. (L) The associations between the methylation status of HCFC1R1 and cg24652919. (M) The associations between the THOC6 and its methylation status. (N) The associations between the THOC6 and cg24652919. (O) The associations between the methylation status of THOC6 and cg24652919. (P) The associations between the P2RY4 and its methylation status. (Q) The associations between the P2RY4 and cg26545968. (R) The associations between the methylation status of P2RY4 and cg26545968. *P < 0.05, **P < 0.01, and ***P < 0.001. [file Image_1.TIF]

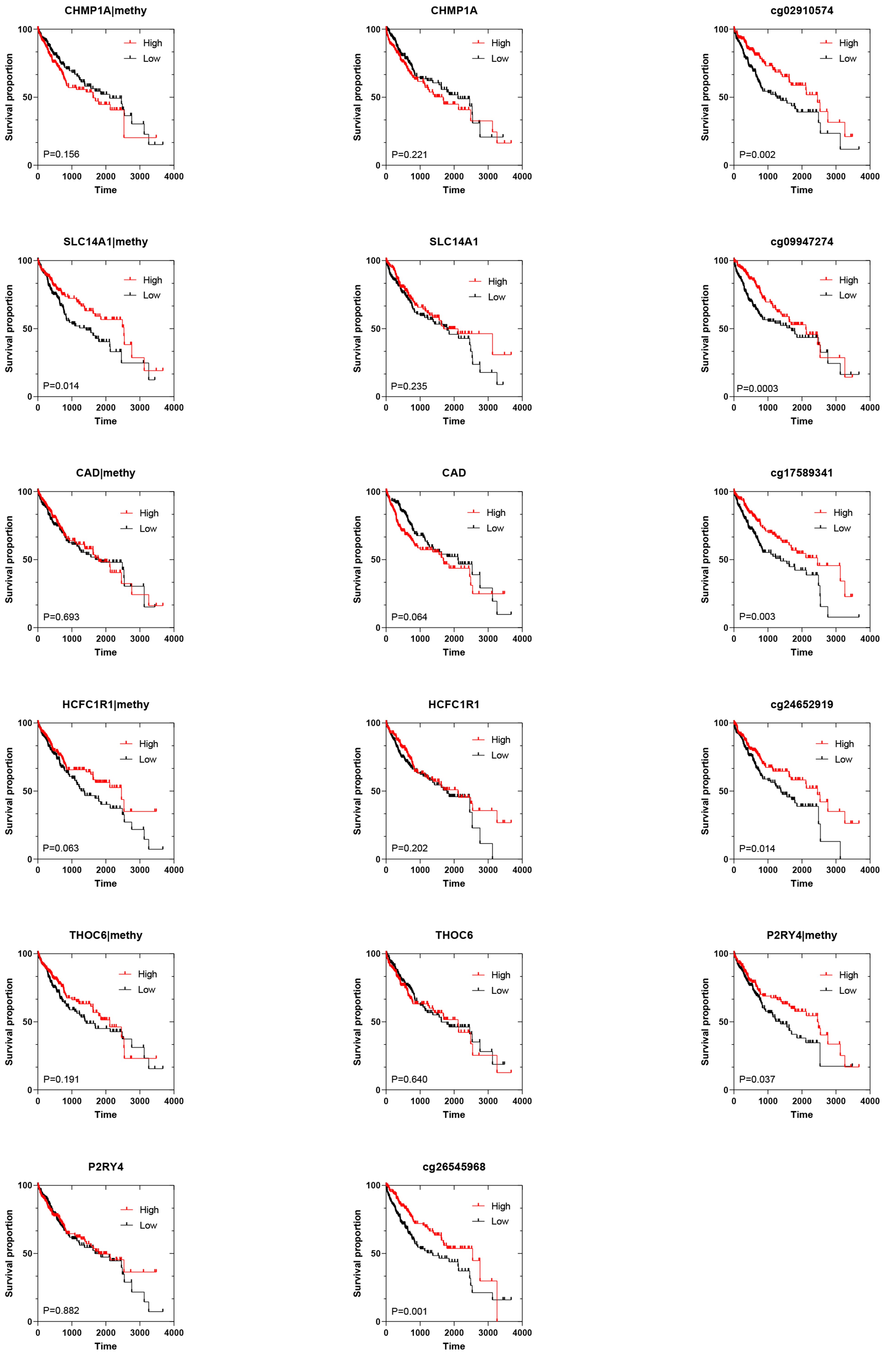

Supplement: Supplemental Figure 2 — Survival analysis was performed to explore the prognostic value of DMCs, their associated genes, and their methylation status; Kaplan–Meier survival curves for each analysis are shown (n = 368 from the LIHC cohort of TCGA). [file Image_2.TIF]

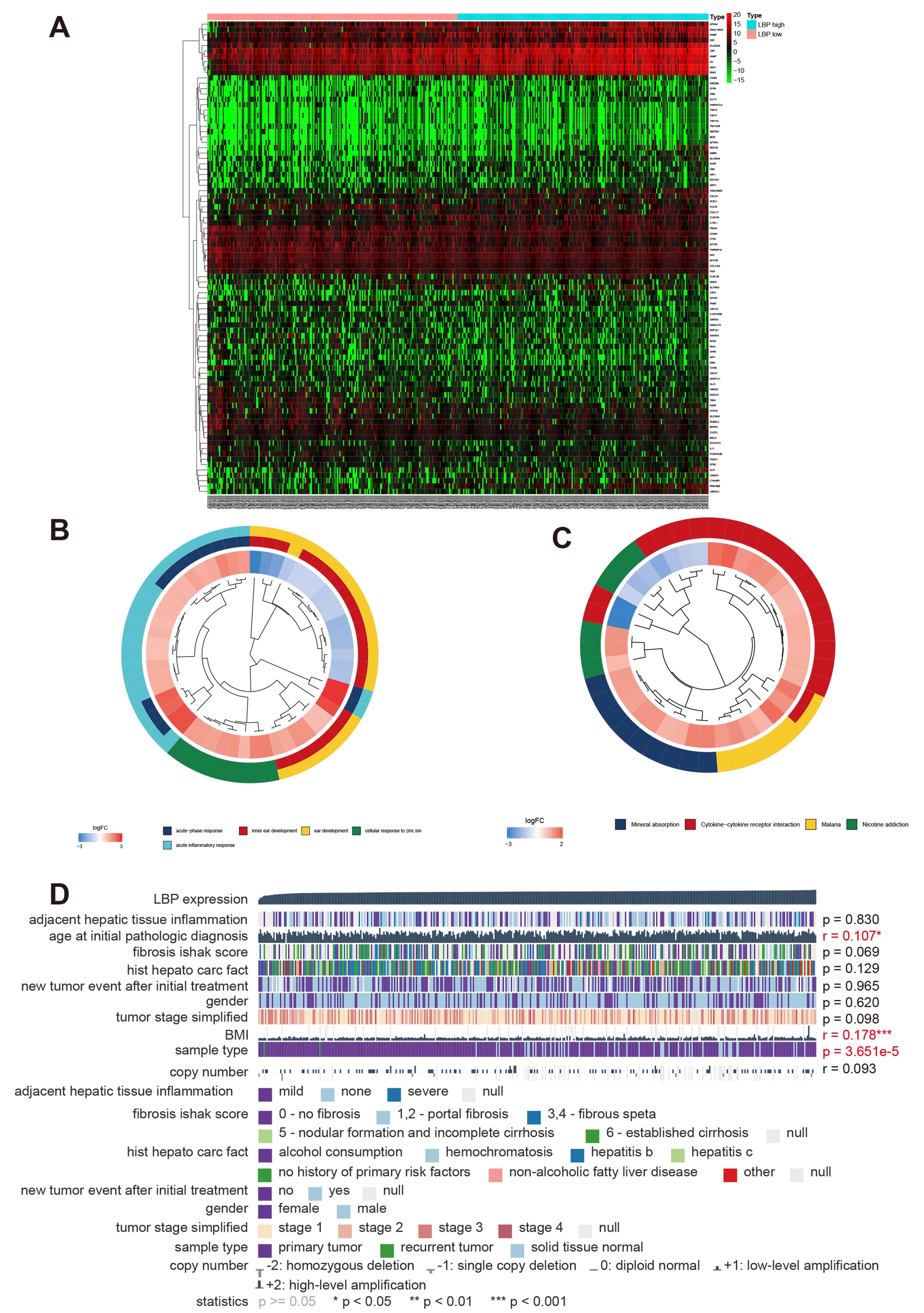

Supplement: Supplemental Figure 3 — Patients from the TCGA cohort were divided into two groups according to the expression of LBP mRNA, and the genes that differed significantly between these two groups are depicted in the heatmap. Rows represent differentially expressed genes, and columns represent samples; green and red colors represent the low and high logFC of gene expression, respectively. Values of P < 0.05 were considered significant (A). The top significantly enriched KEGG pathways and GO terms of differentially expressed genes in HCC. The inner circle represents the logFC of differentially expressed genes, and the red and blue colors represent the high and low logFC values for gene expression, respectively. The outer circle represents the GO terms (B) and KEGG pathways (C) associated with the differentially expressed genes. The expression of LBP mRNA with different clinicopathological features is depicted (D). The numbers on the far right indicate the significance of the correlation (correlation coefficient or P-value, depending on the data types compared) between each row of data (clinical characteristics, expression). *P < 0.05, **P < 0.01, and ***P < 0.001. [file Image_3.JPEG]
