# Supplementary material for: Identification of Aberrantly Methylated Differentially CpG Sites in Hepatocellular Carcinoma and Their Association With Patient Survival
Source: Front Oncol. 2020 Jul 23;10:1031. doi: 10.3389/fonc.2020.01031 (PMC7390903; doi:10.3389/fonc.2020.01031)
Supplement: Supplemental Table 1 — GEO series used in our study. [file Table_1.DOCX]

Supplemental Table 1. GEO Series used in our study.

| Series | Experiment type | Platform | Sample | Year | Country |
| --- | --- | --- | --- | --- | --- |
| GSE37988 | Methylation profiling by array | Illumina HumanMethylation27 BeadChip | 62 pairs | 2012 | USA |
| GSE54503 | Methylation profiling by genome tiling array | Illumina HumanMethylation450 BeadChip | 66 pairs | 2014 | USA |
| GSE57956 | Methylation profiling by array | Illumina HumanMethylation27 BeadChip | 59 pairs | 2014 | Singapore |
| GSE73003 | Methylation profiling by array | Illumina HumanMethylation27 BeadChip | 20 pairs | 2015 | Japan |
