# Supplementary material for: Identification of Aberrantly Methylated Differentially CpG Sites in Hepatocellular Carcinoma and Their Association With Patient Survival
Source: Front Oncol. 2020 Jul 23;10:1031. doi: 10.3389/fonc.2020.01031 (PMC7390903; doi:10.3389/fonc.2020.01031)
Supplement: Supplemental Table 2 — Clinical and pathological characteristics of HCC patients in GSE54503, GSE57956, GSE37988, and GSE73003. [file Table_2.DOCX]

Supplemental Table 2. Clinical and pathological characteristic of HCC patients in GSE54503, GSE57956, GSE37988, and GSE73003.

|  | GSE37988 (n=62) | | GSE54503 (n=66) | | GSE57956 (n=59) | | GSE73003 (n=47) | |
| --- | --- | --- | --- | --- | --- | --- | --- | --- |
| Variables | n | % | n | % | n | % | n | % |
| Gender |  |  |  |  |  |  |  |  |
| Male | 54 | 87 | 50 | 75.8 | 53 | 90 | 37 | 78.7 |
| Female | 8 | 13 | 16 | 24.2 | 6 | 10 | 10 | 21.3 |
| Viral infection |  |  |  |  |  |  |  |  |
| HBV (+) | 49 | 65 | 17 | 24.3 | 36 | 61 | 8 | 17 |
| HCV (+) | 19 | 25 | 23 | 32.9 | /^*^ | / | 25 | 53 |
| other | 7 | 10 | 30 | 42.8 | 23 | 39 | 14 | 30 |
| Cirrhosis |  |  |  |  |  |  |  |  |
| No | / | / | 26 | 39.3 | 37 | 64 | 21 | 44.7 |
| Yes | / | / | 36 | 54.6 | 21 | 36 | 26 | 55.3 |
| Missing | / | / | 4 | 6.1 | / | / | / | / |
| Tumor grade |  |  |  |  |  |  |  |  |
| I-II | / | / | 23 | 34.9 | 28 | 47.5 | 15 | 31.9 |
| III | / | / | 22 | 33.4 | 26 | 44 | 18 | 38.3 |
| IV | / | / | 18 | 27.2 | 5 | 8.5 | 14 | 29.8 |
| Missing | / | / | 3 | 4.5 | / | / | / | / |
| Alcohol drinking |  |  |  |  |  |  |  |  |
| No | 41 | 66 | 27 | 40.9 | / | / | / | / |
| Yes | 8 | 13 | 36 | 54.6 | / | / | / | / |
| Missing | 13 | 21 | 3 | 4.5 | / | / | / | / |
| Cigarette smoking |  |  |  |  |  |  |  |  |
| No | 25 | 40 | 26 | 39.3 | / | / | / | / |
| Yes | 24 | 39 | 36 | 54.6 | / | / | / | / |
| Missing | 13 | 21 | 4 | 6.1 | / | / | / | / |
| Tumor multifocality |  |  |  |  |  |  |  |  |
| Single | / | / | / | / | 45 | 82 | 25 | 53 |
| Multiple | / | / | / | / | 10 | 18 | 22 | 47 |

*/：Not reported or not existed.
