# Supplementary material for: Identification of Aberrantly Methylated Differentially CpG Sites in Hepatocellular Carcinoma and Their Association With Patient Survival
Source: Front Oncol. 2020 Jul 23;10:1031. doi: 10.3389/fonc.2020.01031 (PMC7390903; doi:10.3389/fonc.2020.01031)
Supplement: Supplemental Table 3 — Clinical and pathological characteristics of 147 HCC patients. [file Table_3.DOCX]

Supplemental Table 3. Clinical and pathological characteristics of 147 HCC patients.

| Parameters | Variables | n | % |
| --- | --- | --- | --- |
| Age at diagnosis (Median=51, range 15–82） | ≥ 51 years old | 80 | 54.4 |
|  | ＜ 51 years old | 67 | 45.6 |
| gender | male | 131 | 89.1 |
|  | female | 16 | 10.9 |
| HBV status | positive | 144 | 98 |
|  | negative | 3 | 2 |
| HCV status | positive | 2 | 1.6 |
|  | negative | 145 | 98.4 |
| Differentiation | I-II | 50 | 34 |
|  | III-IV | 97 | 66 |
| TNM | I-II | 119 | 80.9 |
|  | III-IV | 28 | 19.1 |
| Alcohol | absent | 133 | 90.5 |
|  | present | 14 | 9.5 |
| Family history | absent | 142 | 96.6 |
|  | present | 5 | 3.4 |
| ALB | ≥35g/L | 102 | 69.4 |
|  | ＜35g/L | 45 | 30.6 |
| Vascular tumor emboli | absent | 85 | 57.8 |
|  | present | 62 | 42.2 |
| Performance status | 0 | 52 | 35.4 |
|  | 1-2 | 95 | 64.6 |
| liver capsule | absent | 85 | 57.8 |
|  | present | 62 | 42.2 |
