# Supplementary material for: Identification of Aberrantly Methylated Differentially CpG Sites in Hepatocellular Carcinoma and Their Association With Patient Survival
Source: Front Oncol. 2020 Jul 23;10:1031. doi: 10.3389/fonc.2020.01031 (PMC7390903; doi:10.3389/fonc.2020.01031)
Supplement: Supplemental Table 4 — Prognostic factors for overall survival (univariate and stepwise multivariate Cox hazard analysis). [file Table_4.DOCX]

Supplemental Table 4. Prognostic factors for overall survival (Univariate and stepwise multivariate Cox hazard analysis)

| Variables | Univariate | Multivariate |  |
| --- | --- | --- | --- |
|  | p-value | Hazard ratio | p-value |
| Age at diagnosis | 0.372 |  |  |
| Gender | 0.61 |  |  |
| BMI | 0.318 |  |  |
| cg08351331 | <0.0001 | 0.023(0.07-0.73) | <0.0001 |
| LBP mRNA | 0.009 |  |  |
| HBV | 0.402 |  |  |
| HCV | 0.007 |  |  |
| Alcohol history | 0.794 |  |  |
| Family history | 0.144 |  |  |
| Blood platelet count | 0.412 |  |  |
| Thrombocytocrit | 0.5 |  |  |
| Platelet distribution width | 0.984 |  |  |
| Performance status | <0.0001 | 0.561(0.301-0.952) | 0.033 |
| Albumin | 0.02 | 0.943(0.900-0.988) | 0.013 |
| Diameter of tumor | <0.0001 |  |  |
| TNM | 0.023 |  |  |
| Tumor capsule | 0.003 |  |  |
| Vascular cancer embolus | <0.0001 | 0.509(0.312-0.832) | 0.007 |
| Node | <0.0001 |  |  |
| Differentiation (Edmonson) | 0.029 |  |  |
